# The RNA Binding Protein SAM68 Transiently Localizes in the Chromatoid Body of Male Germ Cells and Influences Expression of Select MicroRNAs

**DOI:** 10.1371/journal.pone.0039729

**Published:** 2012-06-22

**Authors:** Valeria Messina, Oliver Meikar, Maria Paola Paronetto, Sara Calabretta, Raffaele Geremia, Noora Kotaja, Claudio Sette

**Affiliations:** 1 Section of Anatomy, Department of Public Health and Cell Biology, University of Rome “Tor Vergata”, Rome, Italy; 2 Laboratory of Neuroembryology, Fondazione Santa Lucia IRCCS, Rome, Italy; 3 Department of Physiology, Institute of Biomedicine, University of Turku, Turku, Finland; 4 Department of Health Sciences, University of Rome “Foro Italico”, Rome, Italy; 5 Digestive and Liver Disease Unit, II Medical School, University of Rome “La Sapienza”, S. Andrea Hospital, Rome, Italy; International Centre for Genetic Engineering and Biotechnology, Italy

## Abstract

The chromatoid body (CB) is a unique structure of male germ cells composed of thin filaments that condense into a perinuclear organelle after meiosis. Due to the presence of proteins involved in different steps of RNA metabolism and of different classes of RNAs, including microRNAs (miRNAs), the CB has been recently suggested to function as an RNA processing centre. Herein, we show that the RNA binding protein SAM68 transiently localizes in the CB, in concomitance with the meiotic divisions of mouse spermatocytes. Precise staging of the seminiferous tubules and co-localization studies with MVH and MILI, two well recognized CB markers, documented that SAM68 transiently associates with the CB in secondary spermatocytes and early round spermatids. Furthermore, although SAM68 co-immunoprecipitated with MVH in secondary spermatocytes, its ablation did not affect the proper localization of MVH in the CB. On the other hand, ablation of the CB constitutive component MIWI did not impair association of SAM68 with the CB. Isolation of CBs from *Sam68* wild type and knockout mouse testes and comparison of their protein content by mass spectrometry indicated that *Sam68* ablation did not cause overall alterations in the CB proteome. Lastly, we found that SAM68 interacts with DROSHA and DICER in secondary spermatocytes and early round spermatids and that a subset of miRNAs were altered in *Sam68^−/−^*germ cells. These results suggest a novel role for SAM68 in the miRNA pathway during spermatogenesis.

## Introduction

Spermatogenesis is characterized by a complex regulation of gene expression. Male germ cells have to face an extended period of silencing of the genome, which occurs during meiotic homologous recombination and during morphological differentiation of haploid spermatids into spermatozoa [1]. As a consequence, a large amount of RNAs is synthesized and stored in male germ cells within ribonucleoprotein particles and granules. The most peculiar example is provided by the chromatoid body (CB), a perinuclear, non-membranous, cloud-like structure that closely resembles the fibrous and dense material named “germ plasm” in lower organisms [2]. In mouse germ cells, few particles resembling the CB first appear in the cytoplasm of late pachytene spermatocytes. After meiosis, the CB condenses to form a single granule in round spermatids, which can be detected until the nucleus of the differentiating spermatids begins to elongate [2]. Due to its large size (∼0.5 μm), the CB can be easily observed by phase contrast microscopy and was first described more than 130 years ago in the cytoplasm of rat spermatids [3]. Nevertheless, its molecular composition and biological function(s) have remained largely unknown for over a century [2], [4], [5]. The recent purification of the CB from mouse germ cells has allowed a more detailed description of its composition [6]. Analysis of the protein content by mass spectrometry revealed that the bulk of the CB mass is composed of six proteins that are involved in different aspects of RNA processing. They include the RNA helicase DDX4/MVH (Mouse VASA Homologue), the PIWI protein named PIWIL1/MIWI, the Tudor domain containing proteins TDRD6 and 7, the gonadotropin regulated testicular helicase DDX25/GRTH and the polyA binding protein PABPC3 [6]. Genetic studies revealed that ablation of *Mvh, Miwi, Grth, Tdrd6* and *Tdrd7* in mice impairs spermatogenesis and male fertility [7]–[11]. Moreover, the CB of round spermatids in *Miwi, Grth, Tdrd6* and *Tdrd7* knockout germ cells exhibits morphological abnormalities [8]–[12]. Although the early meiotic arrest of spermatogenesis in *Mvh* knockout mice prevented this analysis [7], it is likely that MVH also plays a key role in CB assembly and function. Thus, the CB seems to function as an RNA-processing centre in male germ cells, as previously demonstrated for the germ plasm of lower organisms [2].

Pioneer studies using cell labelling and histochemistry had already suggested the presence of RNA and ribonucleoproteins in the CB few decades ago [13], [14]. More recent observations have confirmed that the CB is a site of accumulation of several classes of RNAs, such as microRNAs (miRNAs) [15], PIWI-interacting RNAs (piRNAs) and mRNAs [6]. The finding that polyadenylated mRNAs accumulate in the CB of spermatids [15] together with miRNAs and proteins that are essential for mRNA silencing, further suggests that the CB participates in translational regulation of specific mRNAs. Interestingly, the CB displays a very dynamic nature in germ cells [4], [16], suggesting that it may collect RNAs and proteins from the nucleus and function as a control station where the fate of a given RNA is decided [2]. However, in spite of its highly dynamic nature, very few proteins have been shown to transiently localize in the CB at specific stages of spermatogenesis. One example is provided by the RNA binding protein (RBP) HuR, which accumulates in the CB of early round spermatids [17]. In this work, we report that SAM68 transiently localizes in the CB during the meiotic divisions and in early post-meiotic cells. SAM68 is a KH-type RBP involved in several steps of RNA processing in male germ cells, whose function is essential for male fertility [18]. We previously described that SAM68 regulates the alternative splicing and translation of specific mRNAs in meiotic and post-meiotic germ cells [18]–[20], and that these functions are necessary for the formation of a functional gamete [18]. We now found that, although the major protein components of the CB are correctly recruited in the absence of SAM68, expression of selected miRNAs is altered in *Sam68^−/−^* male germ cells. Together with the observation that SAM68 interacts with DROSHA and DICER, these results suggest a novel role for SAM68 in CB-linked RNA processing events and in the miRNA pathway during spermatogenesis.

## Results

### SAM68 localizes in perinuclear granules in secondary spermatocytes and early round spermatids

**Figure 1:**
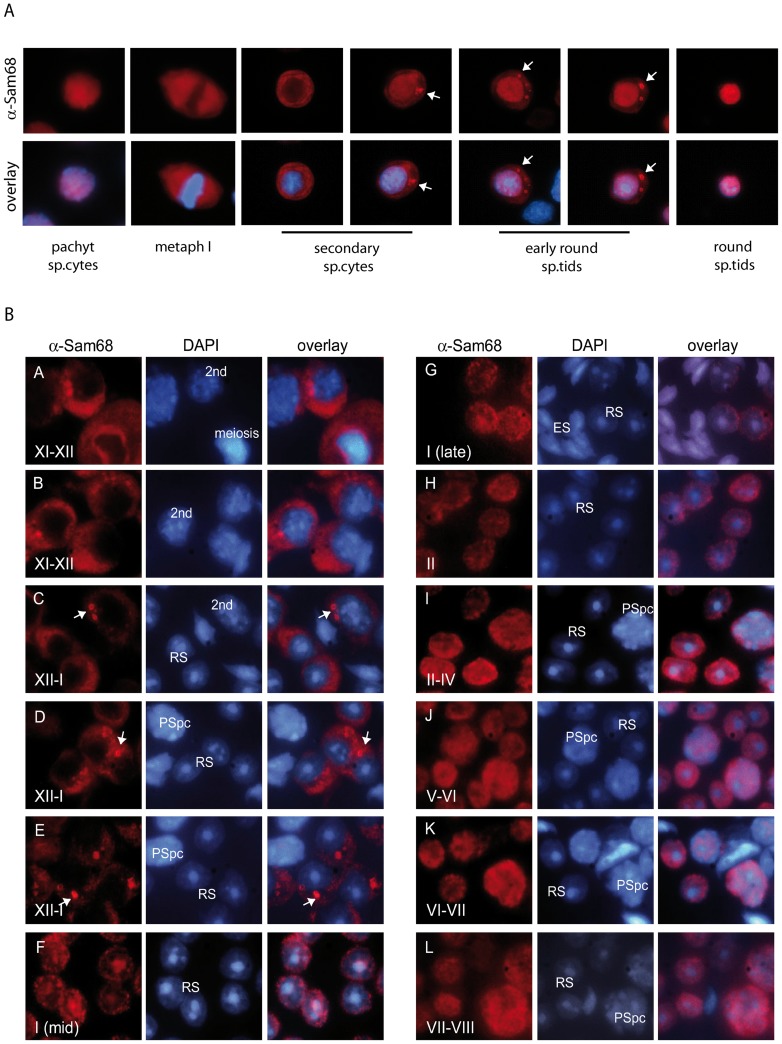
SAM68 accumulates in a perinuclear organelle in secondary spermatocytes and early round spermatids. (**A**) Purified male germ cells were stained with an anti-SAM68 antibody (red) and co-stained with Hoechst (blue) to detect nuclei and to identify cell stages by nuclear morphology. In secondary spermatocytes and early round spermatids SAM68 accumulates into a granule (white arrows) resembling the chromatoid body. (**B**) Stage specific localization of SAM68 during spermatogenesis. Squashes of male germ cells from seminiferous tubules at different stages of spermatogenesis show that SAM68 (red) localizes in the cytoplasm and was enriched in perinuclear granules (arrows) in meiotic spermatocytes from stage XII tubules and in early round spermatids from stages XII and I (A–E). In late stage I spermatids and from stage II through VIII, SAM68 was predominantly nuclear (F–L). Cells were co-stained with DAPI to detect nuclei. 2nd  =  secondary spermatocytes; RS  =  round spermatid; PSpc  =  pachytene spermatocyte; ES  =  elongated spermatid.

SAM68 shuttles between nucleus and cytoplasm in differentiating germ cells [18], [20]. To investigate in more detail the dynamic nature of its localization, we performed immunofluorescence analysis of purified male germ cells. As previously reported [20], SAM68 is localized in the nucleus of pachytene spermatocytes, it translocates into the cytoplasm of secondary spermatocytes and it localizes again in the nucleus of round spermatids (Figure 1A). However, we also observed that SAM68 accumulates in perinuclear, dense granules resembling the CB in secondary spermatocytes and early round spermatids (Figure 1A).

SAM68 was reported to accumulate in cytoplasmic stress granules upon several cellular stresses [21]–[23]. To rule out the possibility that the accumulation of SAM68 in perinuclear granules is caused by stress occurring during the purification procedure, we analysed its localization in germ cells squashed out directly from the seminiferous tubules. This technique allows precise description of the stage of the seminiferous tubule by phase contrast microscopy before fixation [24]. We found that SAM68 localized in the cytoplasm and was enriched in perinuclear granules in meiotic spermatocytes from stage XII tubules and in early round spermatids from stages XII and I (Figure 1B). By contrast, starting from late stage I spermatids, SAM68 was predominantly nuclear and this localization was maintained from stage II through VIII (Figure 1B).

These results indicate that SAM68 transiently accumulates in perinuclear granules during the meiotic divisions and the early phases of spermiogenesis.

### SAM68 co-localizes with MVH and MILI in the chromatoid body of secondary spermatocytes

**Figure 2:**
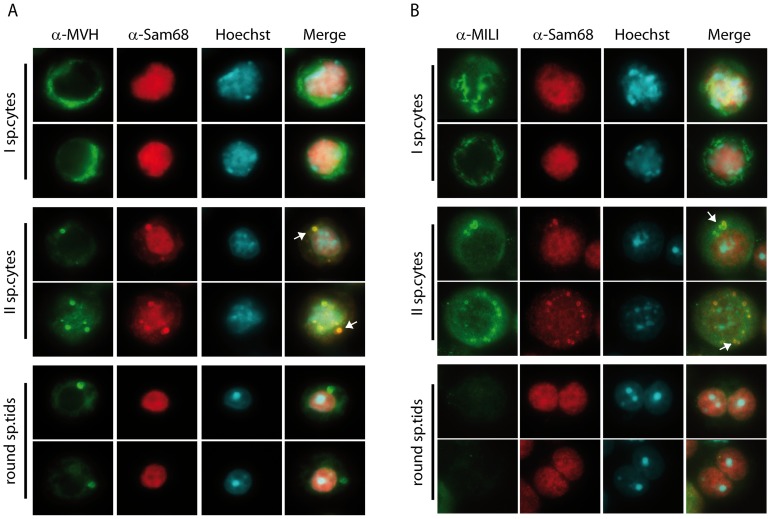
Co-localization of SAM68 with MVH and MILI in male germ cells. (**A**) Isolated male germ cells were co-stained with an anti-SAM68 antibody (red), an anti-MVH antibody (green) and with Hoechst (blue) to detect nuclei. SAM68 and MVH partially co-localize in the CB of secondary spermatocytes (arrows), while in primary spermatocytes SAM68 is nuclear and MVH is cytoplasmic, and in round spermatids SAM68 is nuclear and MVH is predominantly localized in the CB. (**B**) Isolated germ cells were analysed by immunofluorescence using the anti-SAM68 antibody (red) and the anti-MILI antibody (green). Nuclei were stained with Hoechst (blue) to identify cell stages by nuclear morphology. In primary spermatocytes SAM68 localizes in the nucleus, while MILI is cytoplasmic; in round spermatids SAM68 is nuclear and MILI is absent. The localization of the two proteins partially overlaps only in the CB of secondary spermatocytes.

To test whether the SAM68-containing granule is the CB, we co-stained purified male germ cells with anti-SAM68 and anti-MVH antibodies or anti-MILI antibodies. SAM68 and MVH localize to different compartments in primary spermatocytes and round spermatids. Namely, SAM68 was found exclusively in the nucleus of meiotic and post-meiotic cells, whereas MVH accumulated in perinuclear structures of primary spermatocytes and in the CB of round spermatids (Figure 2A). However, in secondary spermatocytes a fraction of SAM68 accumulated in the CB-like granules together with MVH (Figure 2A). Similar results were obtained by comparing the localization of SAM68 with that of MILI [25]. As shown in Figure 2B, MILI localized to fibrous, perinuclear structures in primary spermatocytes and in CB-like perinuclear granules that also contained SAM68 in secondary spermatocytes. By contrast, MILI was absent in the majority of round spermatids whereas SAM68 was localized in the nucleus. These results strongly suggest that SAM68 transiently accumulates in the granules that will form the CB during the second meiotic division and in early round spermatids.

**Figure 3:**
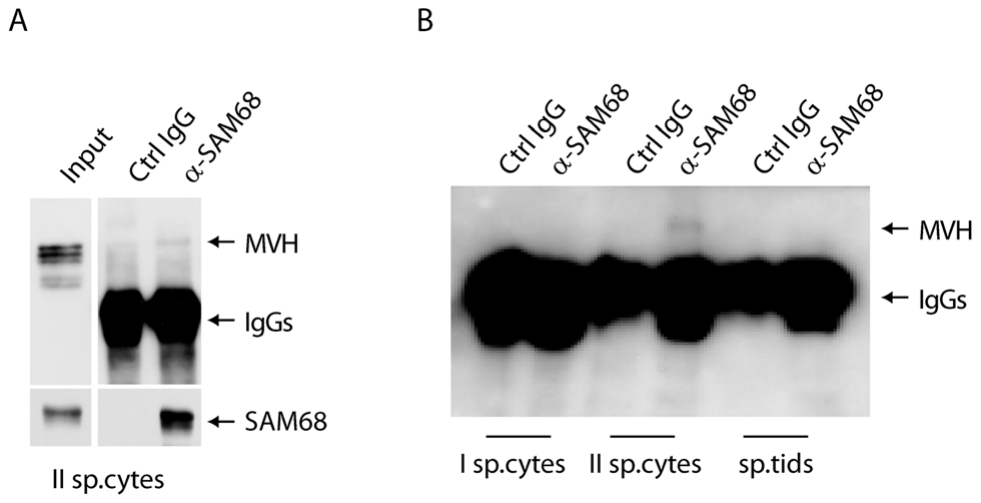
SAM68 co-immunoprecipitates with MVH in secondary spermatocytes. (**A**) Total extract from secondary spermatocytes were immunoprecipitated with an anti-SAM68 antibody and analysed in Westeern blot with anti-MVH and anti-Sam68 antibodies. (**B**) Cellular extracts from spermatocytes (I sp.cytes), secondary spermatocytes (II sp.cytes) and round spermatids (sp.tids) were immunoprecipitated with an anti-SAM68 antibody and detected with anti-MVH antibody. Western blot analysis shows a specific interaction of the two proteins in secondary spermatocytes, while no signal is detected in other germ cell populations.

To corroborate the association of SAM68 with CB components, we performed immunoprecipitation experiments from purified germ cells. Extracts obtained from a germ cell fraction enriched in secondary spermatocytes [20] were immunoprecipitated with anti-SAM68 or control antibodies. Western blot analysis revealed that MVH was co-immunoprecipitated with the anti-SAM68, but not with control IgGs (Figure 3A). Importantly, co-immunoprecipitation was due to a specific interaction in the dividing meiotic germ cells, as it was not detected in primary spermatocytes and round spermatids (Figure 3B), in which the two proteins localize in different cellular compartments (Figure 2A).

### Structural proteins of the chromatoid body are not affected by the ablation of *Sam68*

**Figure 4:**
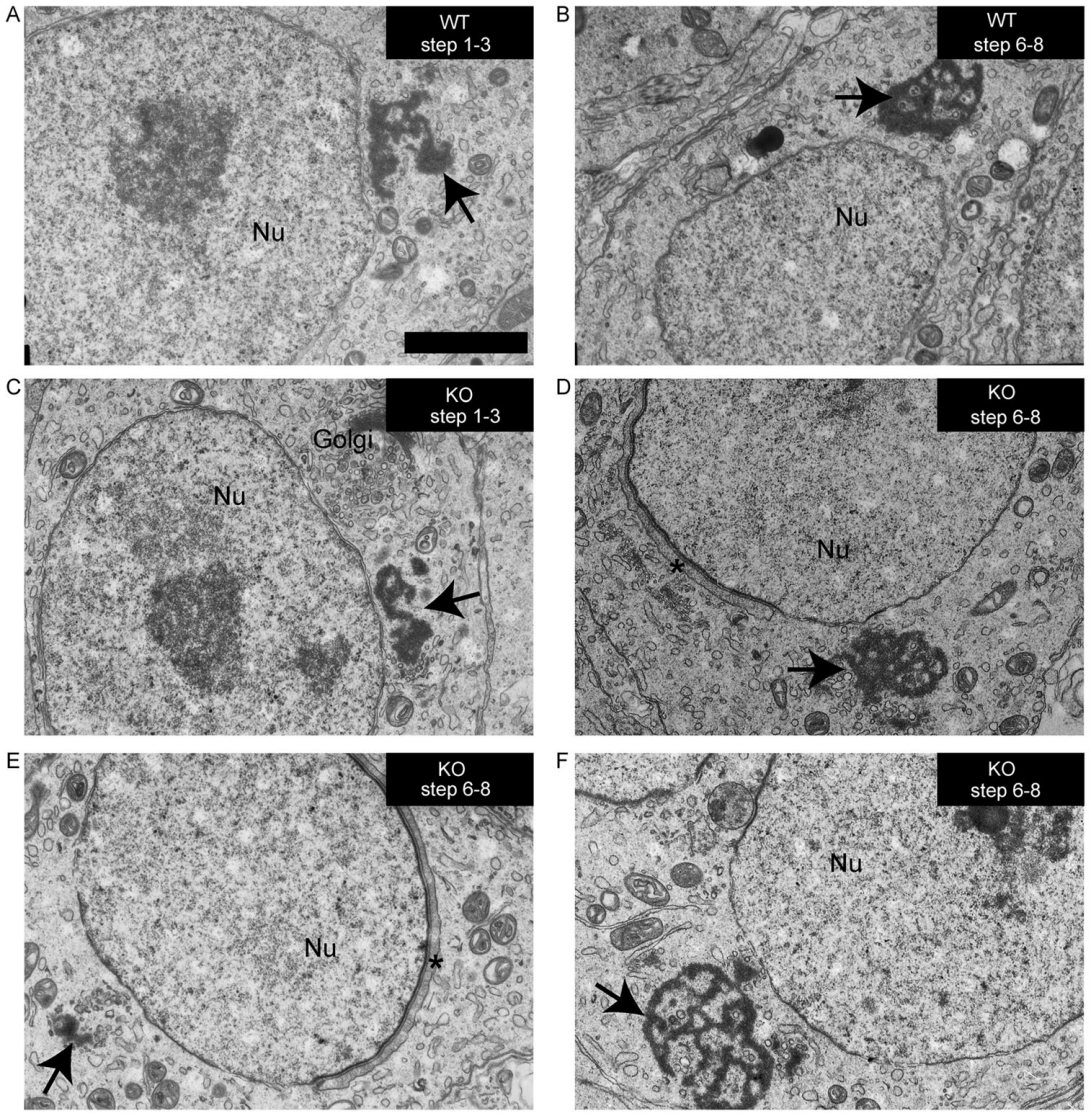
Morphology of the chromatoid body in *Sam68^−/−^* germ cells. (**A, B**) Round spermatids were analyzed by electron microscopy to study the possible changes in the morphology of the CB caused by the deletion of *Sam68* gene. No differences were found between the early chromatoid bodies (steps 1–3 of round spermatid differentiation) in the control (**A**) and knockout (**C**) testes. The knockout CBs appeared condensed and morphologically normal. In late round spermatids (steps 6–8), the knockout CBs appeared mostly normal (**D**) as compared to the control CBs (**B**). Abnormalities in the CB morphology were also commonly observed in *Sam68^−/−^* late round spermatids, such as decreased amount of the chromatoid material (**E**) or excess space between the CB lobes (**F**). Arrows point to the CB. Acrosome is indicated by an asterisk. The genotypes and the steps of spermatid differentiation are shown in the upper right corner of each image. WT, *Sam68^+/+^*; KO, *Sam68^−/−^*; Nu, nucleus. Scale bar is 2 µm.

To further investigate the role of SAM68 in the CB, we subjected sections of *Sam68* wild-type and knockout testes to high-resolution morphological analysis by transmission electron microscopy (EM). We found that the CBs in *Sam68^−/−^* late round spermatids (steps 6–8) were often smaller and/or appeared incorrectly assembled (Figure 4E, F) as compared to those of wild type germ cells, which are characterized by large and dense masses with many clear islands (Figure 4B). However, in some *Sam68^−/−^* late round spermatids the CB appeared normal (Figure 4D). Furthermore, in early round spermatids (steps 1–3), the CBs appeared mostly unaffected (Figure 4A, C).

**Figure 5:**
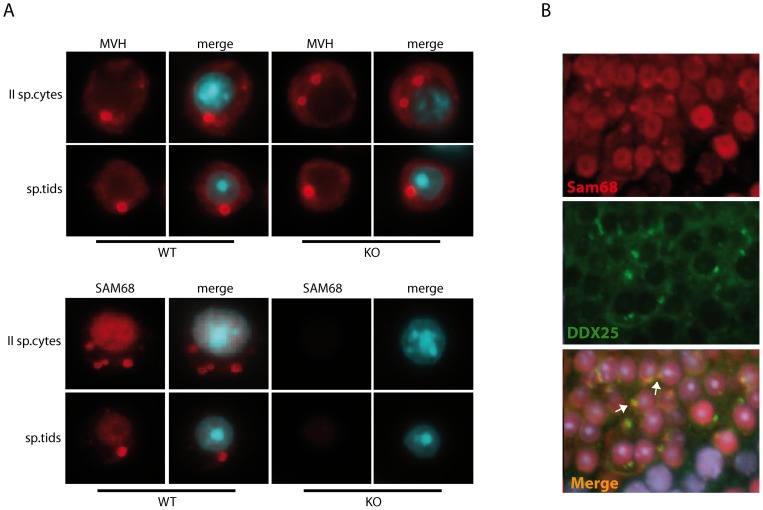
Localization of MVH and SAM68 in the chromatoid body of Sam68 and Miwi knockout germ cells. (**A**) Immunofluorescence analysis with anti-MVH (upper panels) or anti-SAM68 (bottom panels) antibodies of *Sam68* wild type (left panels) and knockout (right panels) male germ cells. Nuclei were detected with Hoechst (blue). (**B**) Immunofluorescence analysis with anti-SAM68 (red) and anti-DDX25 (green) antibodies of *Miwi* knockout (right panels) male germ cells. Nuclei were detected with Hoechst (blue).

Since the *Sam68* knockout testis is characterized by abnormal differentiation and extensive loss of haploid germ cells [18], the impaired morphology of the CB could be a secondary, indirect effect. To test this hypothesis, we checked whether any CB structural component was lacking in the *Sam68* knockout spermatids. First, we determined the localization of MVH by immunofluorescence analysis of wild type and knockout secondary spermatocytes and early round spermatids. As shown in Figure 5A, the absence of SAM68 did not impair MVH localization in the CB. In addition, we also found that ablation of MIWI, a constitutive component of the CB [6], did not impair the localization of SAM68 in the CB (Figure 5B).

**Figure 6:**
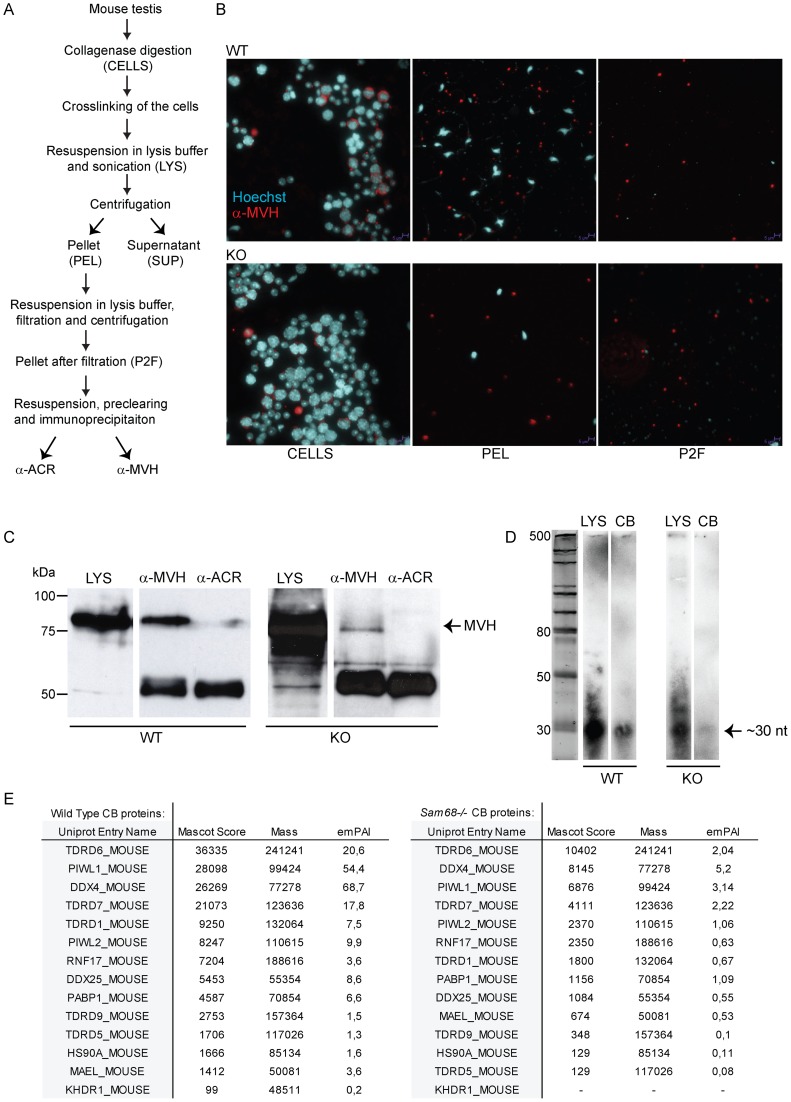
Isolation and analysis of chromatoid bodies from male germ cells. (**A**) Schematic representation of the CB isolation protocol. (**B**) Immunofluorescence analysis of different steps of CB immunoprecipitation experiment, comparing *Sam68* wild type (WT) and knockout (KO) extracts. Cells were stained with an anti-MVH antibody (red) and with Hoechst to detect nuclei. CELLS  =  cells before lysis; LYS  =  lysate; PEL  =  pellet fraction; P2F  =  pellet fraction after filtration. (**C**) Immunoblotting of the CB extracts with anti-MVH antibody to validate the success of the purification. The less intensive signal in the knockout CB fraction indicates the lower number of CBs isolated from the knockout testes compared to the control. anti-ACR, negative control IP; anti-MVH, CB IP. (**D**) RNA gel to demonstrate the presence of piRNAs. Total RNA was extracted from the the lysate (LYS) and CB IPs (CB), radiolabelled and run into a polyacrylamide gel in. (**E**) Mascot analysis of the main CB components. All major CB proteins were present in the knockout CBs. The uniprot entry name KDHR_MOUSE equals to SAM68.

Next, to obtain a comprehensive picture of the CB in *Sam68^−/−^* germ cells, we used a recently developed method to isolate the CB by immunoprecipitation of MVH after crosslinking and lysis of testicular germ cells [6]. The CB isolation protocol is schematically represented in Figure 6A. All the steps of the immunoprecipitation were monitored by immunofluorescence analysis with the CB-specific anti-MVH antibody, which detected a CB-associated granular signal in the pellet fractions (PEL and P2F) (Figure 6B), but not in the supernatant fractions (data not shown). As previously reported [18], the number of round spermatids in the knockout testes was lower than in the wild type testes, thus the isolation yielded less CBs. This is evident from the much weaker MVH signal observed by Western blot analysis in the CB extracts of the knockout samples (Figure 6C). Poly(A)-containing RNAs and small regulatory RNAs such as piRNAs are known to accumulate in the CB [6]. To visualize the small RNA species present in the CBs, total RNA extracted from the control and knockout fractions were 5'-labeled with [γ-32P]ATP and separated on a 15% denaturing polyacrylamide gel. This analysis showed that the *Sam68* ^−/−^ CBs contained the characteristic ∼30 nucleotide (nt) piRNA band (Figure 6D), although in lower quantities probably due to the lower yield of CBs obtained from the knockout sample.

The purified CBs from wild type and knockout germ cells were then subjected to analysis by mass spectrometry. The main constituents of the CB are MVH, MIWI, TDRD6, TDRD7, GRTH and PABPC3 [6]. In particular, four of these proteins (MIWI, GRTH, TDRD6 and TDRD7) are essential in the maintenance of the normal architecture of the CB [2]. All the main components including the Tudor-domain containing proteins (TDRD1, TDRD6, TDRD7), DEAD box helicases (DDX25 and MVH), poly(A)-containing proteins (PABPC1), PIWI proteins (MIWI and MILI) and other proteins associated with piRNA pathways (MAEL) were present in the knockout CBs (Figure 6E). In agreement with the immunofluorescence results, SAM68 was found in the control CBs but not in the knockout CBs. Some less abundant CB proteins present in the control samples were missing from the *Sam68* ^−/−^ CBs (data not shown). However, since the *Sam68* ^−/−^ CB samples were less concentrated, it is likely that less abundant CB components could not be identified reliably.

These results suggest that SAM68 expression is not essential for the CB structure.

### SAM68 ablation affects the expression of a subset of microRNAs in male germ cells

**Figure 7:**
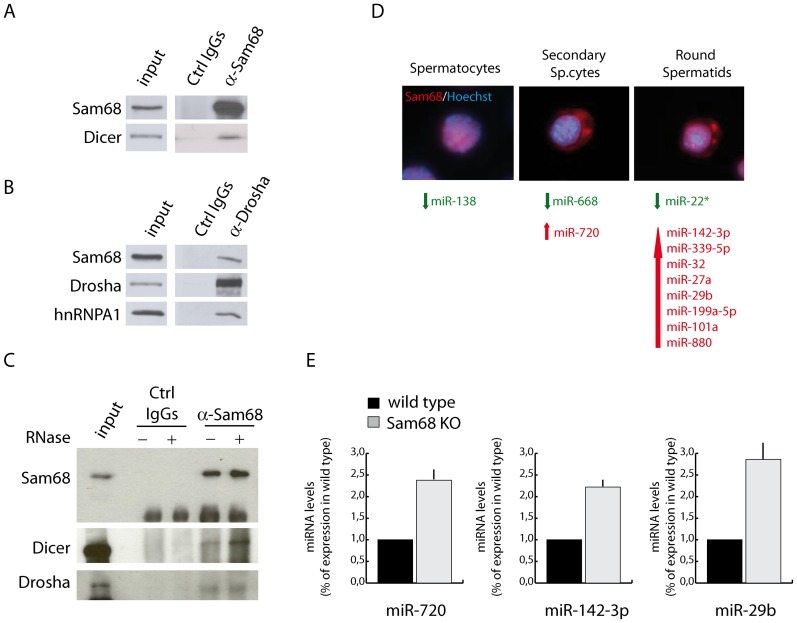
SAM68 is involved in the miRNA pathway in male germ cells. (**A**) Total extract from secondary spermatocytes were immunoprecipitated with an anti-SAM68 antibody and analysed in Western blot with anti-DICER and anti-Sam68 antibodies. (**B**) Cellular extracts from secondary spermatocytes (II sp.cytes) were immunoprecipitated with an anti-DROSHA antibody and detected with anti-DROSHA, anti-SAM68 and anti-hnRNP A1 antibodies. (**C**) Western blot analysis of co-immunoprecipitation performed in the presence of RNase in the cell extract. (**D**) Schematic representation of the microRNAs differentially expressed in the indicated *Sam68* knockout germ cells. In green are indicated the down-regulated microRNAs, in red the up-regulated ones. The immunofluorescence images show the localization of SAM68 in the corresponding wild type germ cells. (**E**) Real Time PCR analysis of miR-720, miR-142-3p and miR-29b in RNA purified from round spermatids of *Sam68* wild-type (black) or knockout (grey) mice. The values were normalized against U6 snRNA and expressed as fold increase with respect to the value of wild type cells.

In addition to PIWI proteins and piRNAs, the CB was also shown to contain miRNAs and proteins involved in the miRNA maturation pathway [15]. Notably, recent studies have documented a role of splicing factors in the biogenesis of selected miRNAs [26]–[29]. To investigate the possible involvement of SAM68, a well known splicing regulator [30], in miRNA biogenesis, we first tested its association with key proteins in the pathway. All miRNAs are initially transcribed as primary transcripts that are successively cleaved by two RNase III enzymes, DROSHA in the nucleus and DICER in the cytoplasm, to produce ∼70 nt long precursor miRNAs and 22 nt long mature miRNAs, respectively [31]. Co-immunoprecipitation experiments indicated that SAM68 interacts with both DICER (Figure 7A) and DROSHA (Figure 7B) in male germ cells. These interaction were not disrupted by treatment with RNase, indicating that they were not mediated by a bridging RNA (Fig. 7C). These results suggest that SAM68 may participate to miRNA biogenesis. To test this hypothesis, we performed a microarray analysis of miRNAs purified from wild type and knockout primary spermatocytes, secondary spermatocytes and round spermatids (Figure 7D). Hybridization of the purified RNAs to the miRCURY LNA microarray revealed that *Sam68* ablation selectively affected 12 miRNAs, the majority of which (9 out of 12) were upregulated in knockout germ cells (Figure 7D). Interestingly, with the exception of miR-138, all these miRNAs were differentially expressed at stages that are concomitant (secondary spermatocytes) or subsequent (round spermatids) to the association of SAM68 with the CB (Figure 7D). To validate the results of the array, we analyzed the expression of three of these miRNAs (miR-720, miR-142-3p and miR-29b) by quantitative real time PCR. As shown in Figure 7E, all of them were upregulated in *Sam68^−/−^* germ cells with respect to the corresponding wild type cells.

These results suggest that SAM68 is involved in the miRNA pathway of male germ cells.

## Discussion

The molecular nature and the function of the CB in germ cells have remained elusive and debated for years [2], [4], [5], [32]. Recently, the demonstration that many RBPs and RNA species associate with this structure, has lead to the general consensus that the CB functions as an RNA-processing centre [2], [5]. The CB material appears in late pachytene spermatocytes and it fuses together to mould into a single structure in each round spermatid after meiosis [2]–[4], [32], [33]. During spermiogenesis, the CB increases in size until step 6, then it moves caudally to the neck region and gradually decreases in size until it disappears in elongating spermatids [5], [14], [33], [34]. Furthermore, the CB dynamically moves within the cytoplasm and exhibits continuous changes in shape and size [4], [14]. Nevertheless, very few examples of proteins that transiently associate with the CB have been reported [2], [16]. In the present study we demonstrate that SAM68 is one of such proteins, which transiently associates with the CB of secondary spermatocytes and early round spermatids.

SAM68 is a ubiquitously expressed KH-type RBP, whose expression levels and localization vary during cell differentiation in several tissues [35]. In particular, in male germ cells SAM68 is mainly nuclear, but it translocates into the cytoplasm of secondary spermatocytes, where it regulates the translation of specific mRNA targets [18], [20]. This function of the protein is essential for the production of a functional gamete, as *Sam68^−/−^* haploid germ cells express lower levels of a subset of proteins involved in cell differentiation, display abnormal morphology and undergo massive cell death through apoptotic pathways [18]. We have now observed that during its translocation in the cytoplasm, SAM68 accumulates in perinuclear structures. Co-staining analysis with both anti-MVH and anti-MILI antibodies confirmed that these granules correspond to the forming CB in meiotic and post-meiotic male germ cells. However, SAM68 expression does not seem to be required for CB assembly and structure. Our EM analysis showed that the CB of *Sam68* ^−/−^ early round spermatids (step-2–3) is normal. In late round spermatids (steps 6–8) from knockout testes we often observed smaller and/or incorrectly assembled CBs as compared to those of wild type germ cells. Nevertheless, it is likely that these abnormalities are indirect consequences of the morphological and molecular defects occurring in *Sam68* ^−/−^ haploid germ cells, which ultimately lead to apoptosis (18). This interpretation is also supported by our mass spectrometry analysis of purified CBs. We found that, although less material was obtained from *Sam68* ^−/−^ testes, the main CB protein constituents were all correctly recruited in the absence of SAM68. This unbiased analysis also confirmed that SAM68 was present in the CB purified from wild type germ cells, thus corroborating the immunofluorescence results. Our study also showed that some of the less abundant CB proteins were absent in the CBs of knockout samples, whereas piRNAs were present in lower amounts. However, since a general lower yield in CB material was obtained from the knockout germ cells, we suggest that these differences are mainly attributable to the lower concentration of these proteins than to their selective differential recruitment.

Several reports have recently linked the CB to components of the miRNA pathway [2]. First, it was shown that DICER, AGO2, AGO3 and some miRNAs were enriched in the CB of round spermatids [15]. Furthermore, ablation of expression of TDRD6, a CB architectural protein, affected the expression of several miRNAs in the testis, suggesting that TDRD6 is involved in the miRNA pathway [10]. More recently, the RNA DEAD-box helicase GEMIN3 (DDX20), a microRNA biogenesis factor, was shown to accumulate in the CB of human and mouse germ cells [36]. On the other hand, many RBPs that play key role in pre-mRNA splicing, like SAM68, have been recently reported to participate to various steps in the processing of miRNAs [26]–[29]. Thus, we directly investigated whether SAM68 is involved in miRNA biogenesis in male germ cells. Indeed, we found that SAM68 interacts with DROSHA and DICER, the two RNase III enzymes involved in the nuclear and cytoplasmic processing of the miRNA precursors [31]. The contribution of Sam68 in the processing of miRNAs was also supported by the changes in selected miRNAs observed in *Sam68^−/−^* male germ cells. Twelve miRNAs were differentially expressed between wild-type and knockout germ cells. The majority of them were up-regulated, whereas only three miRNAs were down-regulated in *Sam68^−/−^* germ cells. Notably, a similar increase of more than 50 miRNAs was also observed in *Tdrd6* knockout testis [10], suggesting that SAM68 may play a similar role in this pathway. The function of most of the SAM68-modulated miRNAs is largely unknown. However, miR-29b was previously proposed to play a role in the modulation of genomic DNA methylation in female primordial germ cells, by negatively regulating the expression DNMT3a and DNMT3b [37]. Notably, miR-29b belongs to a family of miRNAs that are often down-regulated in human cancers, including hormone-resistant prostate cancer (PCa) [38], [39], and increased ectopic expression of miR-29b suppressed the metastatic phenotype of PCa cells by repressing epithelial-mesenchymal transition (EMT) signalling [39]. Since SAM68 is frequently up-regulated in PCa, where it contributes to cell proliferation and survival [40], [41], these observations suggest that it might regulate miR-29b also in cancer cells. In line with this hypothesis, recent observations indicate that SAM68 contributes to oncogenic transformation by promoting EMT [42]. Thus, it is possible that increased expression of SAM68 in PCa cells is required to suppress miR-29b expression, hence contributing to the aggressive phenotype of these cells.

The expression of miRNAs can be regulated at the post-transcriptional level by modulating nuclear and cytoplasmic processing events. For instance, the KH-type splicing regulatory protein KSRP binds to the terminal loop of its target miRNAs, thereby promoting their processing [28]. The function of KSRP is antagonized by another splicing factor: hnRNP A1. It was shown that hnRNP A1 competes with KSRP for binding to the conserved terminal loop of pri-let-7a-1 and inhibits its processing by DROSHA [27]. Interestingly, hnRNP A1 interacts with DROSHA [26] and with SAM68 [43] in somatic cells, and we found that these three proteins can be co-immunoprecipitated together in male germ cell extracts (Figure 7B). Noteworthy, SAM68 and hnRNP A1 cooperate in the regulation of splicing of at least two common targets [43], [44]. Thus, although direct studies to test this possibility need to be performed in the near future, it is tempting to speculate that SAM68 participates to hnRNP A1-mediated processing of selected miRNAs.

In conclusion, our study identifies SAM68 as a novel CB component that transiently associates with this structure and provide evidence for the involvement of SAM68 in the miRNA pathway during spermatogenesis.

## Materials and Methods

### Cell isolation and culture

The *Sam68* knockout colony was maintained by crossing *Sam68* heterozygous mice. Genotyping of the mice was performed as described previously [45]. Testes from 40–60 day-old CD1 mice (Charles River Laboratories) and C57BL6 *Sam6*8 wild-type and knockout mice were used to obtain pachytene spermatocytes, secondary spermatocytes, and round spermatids by elutriation technique as described previously [20], [46]. After elutriation, pachytene spermatocytes were cultured in minimum essential medium (Invitrogen) and supplemented with 0.5% BSA (Sigma- Aldrich), 1 mM sodium pyruvate, and 2 mM lactate at 32°C in a humidified atmosphere containing 95% air and 5% CO2. At the end of the incubation, cells were washed in phosphate buffered saline (PBS) and used for further experiments.

### Immunofluorescence analysis

Mouse germ cells were fixed in 4% paraformaldehyde (PFA) and washed three times in PBS. Cells were permeabilized with 0.1% Triton X-100 (Sigma-Aldrich) for 10 min and then incubated for 1 h in 0.5% BSA. Cells were washed three times in PBS and incubated for 2 h at room temperature (RT) with antibodies against SAM8 (1∶1000, Santa Cruz Biotechnology), MVH (1∶500; Abcam) or MILI (1∶200, Abcam), followed by 1 h incubation with cy3-conjugated anti-mouse IgGs (Alexa Fluor) or FITC-conjugated anti-rabbit IgGs (Alexa Fluor). After washes, slides were mounted with Mowiol reagent (EMD) and analyzed by microscopy at RT as described [22], [47]. Images were taken from a fluorescent microscope (Axioskop; Carl Zeiss, Inc.) using a Pan-Neofluar 40×/0.75 objective lens, and from an inverted microscope (DMI6000B; Leica) using a Pan-Neofluar 40×/0.75 objective lens. Images were acquired as TIFF files using an RT slider camera (Diagnostic Instruments) and the IAS2000 software (Biosistem 82) or LIF software (Leica). Photoshop and Illustrator softwares (Adobe) were used for composing the panels.

### Immunoprecipitation experiments

Isolated mouse germ cells were washed in PBS and homogenized in lysis buffer [100 mM NaCl, 10 mM MgCl2, 30 mM Tris-HCl, pH 7.4, 1 mM DTT, and protease inhibitor cocktail (Sigma-Aldrich)] supplemented with 0.5% Triton X-100. Soluble extracts were separated by centrifugation at 10000 *g* for 10 min, pre-cleared for 2 h on protein A–Sepharose beads (Sigma-Aldrich) in the presence of rabbit IgGs (5 μg) and 0.05% BSA. After centrifugation for 1 min at 1000 *g*, supernatant fractions were incubated with rabbit anti-SAM68, anti-DROSHA (Abcam) or control IgGs (5 μg) for 3 h at 4°C under constant rotation. After washing, the beads were eluted in SDS sample buffer for Western blot analysis with anti-MVH antibody (1∶500).

### Chromatoid body immunoprecipitation

Testes from adult C57BL6 *Sam68* wild-type and knockout mice were digested in PBS containing 0.5 mg/ml of collagenase for 60 minutes at room temperature. Digested tubules were filtered through 100 μm filter (BD biosciences) and then centrifuged for 5 minutes at 300 *g*, 4°C. After two washes in PBS, cells were crosslinked in 0.1% PFA for 20 min. The reaction was stopped by adding 0.25 M glycine, pH 7. Cells were washed in PBS, resuspended in RIPA buffer [150 mM NaCl, 50 mM Tris-HCl, pH 7.5, 1 mM DTT, 1% Nonidet P40, 0.5% sodium deoxycholate, 1∶200 Riboblock (Fermentas) and protease inhibitor cocktail (Roche)] and sonicated with Bioruptor UCD-200 sonicator (6 cycles of 30 s with 30 s intervals). The cell lysate was centrifuged for 10 minutes at 500 *g*. The pellet containing the CBs was filtered and resuspended in RIPA buffer and used for immunoprecipitation [6]. Cell lysates were pre-cleared with Dynabeads protein G (Invitrogen) for 30 minutes, and then added to Dynabeads protein G coupled with rabbit polyclonal anti-MVH antibody (Abcam, ab13840) or rabbit-polyclonal anti-ACROSIN (Santa Cruz, sc-67151) antibody. Tubes were incubated overnight at 4°C under rotation. Beads were rinsed, resuspended in RIPA buffer and the crosslinks were reversed in the same buffer (for RNA analysis) or in SDS sample buffer (for protein analysis) at 70°C for 45 minutes.

### Electron microscopy

Small pieces of testis were cut, fixed in 5% glutaraldehyde and treated with a potassium ferrocyanide-osmium fixative. The samples were embedded in epoxy resin (Glycidether 100, Merck), sectioned, stained with 5% uranyl acetate and 5% lead citrate, and visualized on a JEOL 1200 EX transmission electron microscope.

### Mass spectrometric analysis

Proteins were shortly separated with NuPAGE Novex Gel System (Invitrogen) followed by in-gel digestion with Trypsin (Promega) and the extraction of peptides. The peptides were analyzed with LC-MS/MS using an Agilent 1200 series nanoflow system (Agilent Technologies) connected to a LTQ Orbitrap mass-spectrometer (Thermo Electron, San Jose, CA, USA) equipped with a nanoelectrospray ion source (Proxeon, Odense, Denmark).

### Microarray analysis

RNA from isolated male germ cells (spermatocytes, secondary spermatocytes and round spermatids) from adult *Sam68^+/+^* (n = 4) and *Sam68^−/−^* (n = 4) mice was extracted kit (QIAGEN). RNA samples were tagged for labeling with Cy3 and Cy5 dyes, respectively using QIAzol lysis reagent and the MIRNeasy mini, by using the miRCURY Labeling Kit (Exiqon) for use with Exiqon miRCURY LNA Array (v 10.0). The array consists of probes for the mature forms of all miRNA present in the miRBase 14.0 release of the miRNA registry. Probes corresponds to 1891 mature miRNAs, each represented twice on the microarray. The locked-nucleic acid miRNA probes are highly sensitive and optimized to minimize cross-hybridization between similar mature miRNAs. Two biological replicates for each sample were analysed. For each sample, 4 μg of total RNA was labeled and hybridized to the microarray. Sample RNA quality control was performed using Bioanalyser2100. Microarray data were normalized using the global Lowess (Locally Weighted Scatterplot Smoothing) regression algorithm (Exiqon).

### Real time PCR

Validation of selected miRNA expression levels was performed using the Taqman® miRNA Expression Assays (Applied Biosystem). RNA was reverse-transcribed using specific miRNA stem-loop primers and the Taqman® miRNA reverse transcription kit (Applied Biosystems). Mature miRNA expression was measured with Taqman® microRNA assays (Applied Biosystems) according to the manufacturer's instructions and normalized for the expression level of the snRNP U6 RNA.
